# Using High-Fidelity Simulation to Introduce Communication Skills about End-of-Life to Novice Nursing Students

**DOI:** 10.3390/healthcare8030238

**Published:** 2020-07-29

**Authors:** Rebeca Abajas-Bustillo, Francisco Amo-Setién, Mar Aparicio, Noelia Ruiz-Pellón, Rosario Fernández-Peña, Tamara Silio-García, César Leal-Costa, Carmen Ortego-Mate

**Affiliations:** 1 Faculty of Nursing, University of Cantabria, 39008 Santander, Spain; rebeca.abajas@unican.es (R.A.-B.); apariciomm@unican.es (M.A.); ruizpeln@gmail.com (N.R.-P.); roser.fernandez@unican.es (R.F.-P.); tamara.silio@unican.es (T.S.-G.); carmen.ortego@unican.es (C.O.-M.); 2 Faculty of Nursing, University of Murcia, 30100 Murcia, Spain; cleal@um.es

**Keywords:** education nursing, students nursing, patient simulation, health communication, end-of -life care

## Abstract

*Background*: High-fidelity simulation is being considered as a suitable environment for imparting the skills needed to deal with end-of-life (EOL) situations. The objective was to evaluate an EOL simulation project that introduced communication skills to nursing students who had not yet begun their training in real healthcare environments. *Methods*: A sequential approach was used. The “questionnaire for the evaluation of the end-of-life project” was employed. *Results*: A total of 130 students participated. Increasing the time spent in high-fidelity simulation significantly favored the exploration of feelings and fears regarding EOL (t = −2.37, *p* = 0.019), encouraged dialogue (t = −2.23, *p* = 0.028) and increased the acquisition of communication skills (t = −2.32, *p* = 0.022). *Conclusions*: High-fidelity simulation promotes communication skills related to EOL in novice nursing students.

## 1. Introduction

Death is a universal and unavoidable event that creates a wide range of emotions and fears. Nurses are not exempt from these feelings [1,2], and many experience difficulties when facing the death of those they have cared for [3,4,5]. Thus, it is important for them to acquire skills during their training to cope with these situations [6,7,8,9,10]. However, this task is not simple. Death is one of the most stressful situations experienced by nursing students [11,12,13,14,15], and there is a need to rely on educational programs. These allow end-of-life care (EOL) skills to be developed in realistic, rather than idealistic, situations, which better prepares them for their professional life.

Several studies have emphasized the importance of health professionals being equipped with strategies and attitudes aimed towards coping with death [16,17,18]. However, training in communication and behavioral skills regarding death is commonly neglected in undergraduate training programs [12,19]. Carmack and Kemery [20] suggest that end-of-life education developed for nursing students should include didactic learning elements, as well as clinical experiences and simulations, including a focus on interprofessional education.

Traditional teaching in the health sciences is based, mainly, on learning technical skills. Non-technical skills, such as leadership, teamwork or communication skills, have not been included in the curriculum. In this sense, high-fidelity simulation is a learning tool that allows the development, in a safe environment, of not only technical skills, but also non-technical skills. In the last 20 years, simulation has evolved remarkably. High-fidelity simulation allows the re-enactment of real situations that include procedures with communication skills, making it a training strategy that facilitates EOL education for health professionals and students [21,22,23,24,25,26,27]. Based on personal experience and the relevant nursing literature, opportunities to reflect on and converse about death is necessary, especially for students who have not yet had experiences in actual healthcare settings. In 2018, Allen [28] conducted a study to evaluate the level of stress of 159 nursing students who participated in an end-of-life simulation. The study obtained more statistically significant measurements of psychological stress when using high-fidelity simulation than when role-playing was utilized. Mileder et al. [29] suggest offering specialized training, focused on relevant psychological aspects, before employing the simulation.

Reflection is a key element in the educational process. The emotional management of the feelings and ideas created when undergoing a process of reflection about death is considered a skill that the students must acquire during their training [13,30]. Schön [31], one of the most influential authors in the development of reflective learning, recommends that the professor, as well as the students, should reflect on their practices, which would re-enforce their learning. David Kolb [32], who developed the Experiential Learning Method (ELM), states that learning is not possible without reflection.

Based on this, we designed and implemented this EOL simulation project intended for new nursing students, which allows for a progressive approach to the subject of death. It aims to promote sensitivity, reflection, dialogue, and the acquisition of communication and behavioral skills for facing death and dying. It uses a dynamic approach into which reflective learning, high-fidelity simulation and Information and Communication Technologies (ICTs) are integrated. The objective of this study was to evaluate an EOL simulation project that introduced communication skills to nursing students who had not yet begun their training in real healthcare environments.

## 2. Materials and Methods

A sequential approach was used to evaluate an EOL simulation project. A total of 130 2nd-year nursing students of the Nursing Degree at the University of Cantabria (Spain), who had not initiated their training in real healthcare settings at the start of the project (although they did have didactic and skills training in nursing care), participated in the project. All students agreed to participate (*n* = 134), but four of them did not provide data.

Students who agreed to participate were divided into four groups of 15–20 individuals and received a three-month educational intervention, consisting of six sessions (Table 1) guided by two to three professors with experience in reflective learning, group dynamics, behavioral assessment and high-fidelity clinical simulation.

SESSION 1—PRESENTATION (30 min): At the beginning of the session, the educational program is shown to the students and their consent is requested. Students who agree to participate are asked to read the information sheet and sign the informed consent.

SESSION 2—REVIEW OF THEORICAL CONCEPTS (60 min): This session is intended to allow practice of the basic theoretical concepts related to active listening and emotional skills.

SESSION 3—APPROACH AND REFLECTION (120 min): The aim of this session is to promote an approach to the subject of death, and to encourage sensitivity and reflection through the reading of children’s stories whose themes revolve around death.

For the development of this session, the reflective learning model by McDrury and Alterio [33] was followed, as it relies on stories to teach the students to reflect on a specific subject. A total of 30 stories (Table A1) were made available to the participants, and they were allowed to freely choose and read one of them.

SESSION 4—DIALOGUE (60 min): The aim of this session is to encourage dialogue on death and the grieving process, following the structure of the death cafe [34]. A death cafe is an informal meeting in which a group of people comes together to talk about death while sharing coffee, cake and a set of questions aimed at promoting dialogue and social awareness about death. The session opened by offering the chance to choose a “menu” which contained 4–5 questions related to death and the dying process, which the students could discuss.

SESSION 5—SIMULATION (120–360 min): The objective of this session is to allow the practice of communication and emotional skills when facing death and dying in a safe environment, such as the simulation classroom.

The simulation sessions were developed in the high-fidelity simulation center of the faculty of nursing of the University of Cantabria, which has a control room, a debriefing room and two simulation rooms. The high-fidelity mannequin used was a SimMan from Leardal^®^ (Madrid, Spain). The simulation center is equipped with audio and video integrated, and the simulation rooms could be modified to simulate a hospital room, an intensive care unit, an operating room or an out-of-hospital scenario. One or two students participate in each scenario, while the rest are in the debriefing room watching the simulation in real time on two screens. Professors or other students (following the professor’s instructions) also participate in the simulation as family members, friends or hospital staff, to make the simulation as realistic as possible. The simulation session is led by a professor from the control room, who is also “the voice and the soul” of the mannequin. Scenarios are recorded, and once the simulation is finished all students meet in the debriefing room. Then, the most relevant moments of the simulation are shown again on the screens. After that, students are asked about their feelings, emotions, decisions and actions following a semi-structured script to guide the debriefing.

During the academic year 2018–2019, 120 min were dedicated to high-fidelity simulation, which was tripled in the academic year 2019–2020 in order to follow the suggestions of most students in the academic year 2018–2019, who recommended that the time dedicated to the high-fidelity simulation be increased for the subsequent courses.

SESSION 6—EVALUATION (60 min): The aims of this last session are twofold: (1) for teachers to evaluate students’ communication skills in simulated EOL situations, and (2) for students to evaluate the educational program and offer suggestions for improvement.

Three teachers evaluated the communication skills of the students when faced with the different EOL situations posed in the simulation scenarios, for which the Gap–Kalamazoo Communication Skills Assessment Form [35] was used, which allows the scoring of the team’s communication on a scale from 1 (poor) to 5 (excellent), in 9 domains. The instrument’s results can be evaluated via individual domains and via general communication score (range 9–45). Higher scores reflect better team communication. Evaluators ranked the top three and worst domains by team, and listed the team’s strengths and weaknesses.

In order for the students to be able to evaluate the project, the questionnaire for the evaluation of the end-of-life project was used. This questionnaire was designed ad hoc for its use in the study, based on the prior literature on the subject; it contains open- and closed-ended questions.

In addition, a discussion group was held in which 11 students participated. The script is shown in Table A2. The focus group session was recorded for audio and video, and lasted 120 min. Once the information was obtained, a literal transcription of the recorded audio material was made and analyzed.

### 2.1. Analysis

For the statistical analysis, the software program IBM SPSS Statistics^®^ v.22 (IBM, Armonk, NY, USA) was utilized. Statistical significance was considered when *p* < 0.05.

The reliability, defined as internal consistency, was analyzed via the Cronbach’s Alpha (α) for each of the dimensions of the instruments.

The validity of the content was found by calculating the content validity index (CVI). The CVI varies from +1 to −1, with positive scores indicating a greater content validity [36].

To estimate if there were significant differences between the closed-ended questions, Chi-squared test was employed.

In order to estimate whether there were significant differences between the evaluation scores for the 2018–2019 vs. 2019–2020 academic year, a t-test for independent samples was applied. Before this, the assumption of normality of the variables was verified through the Kolmogorov–Smirnov test.

The qualitative content analysis of the information from the focus groups was conducted inductively, to allow for the emergence of indicators and categories from the data, taking into consideration the thematic structure of the scripts followed. Although this thematic deductive structure served to organize the initial work, the emergence of ideas from the data shaped the ideas of the study.

The qualitative content analysis performed is composed of different analytical cycles [37,38,39]. In the first place, an initial substantive coding was performed next to the data, which was complemented by coding in real time, to track the metaphors in the discourse of the participants or the terms with a high degree of significance. In the second place, a process of categorization and re-coding was performed, which placed the different indicators into different categories or subjects at the same time as a system of coding was being reviewed and filtered, allowing for work with a greater level of abstraction.

### 2.2. Ethical Considerations

The permissions needed for conducting the intervention were obtained (CE Project 01/2018). The students were informed about the aim of the study, and gave their informed consent to voluntarily participate in it, expressively authorizing their being recorded during the session.

## 3. Results

A total of 130 second-year students enrolled in the Nursing Degree participated in the study. The mean age was 20.7 years (SD = 4.8), 86.9% (*n* = 113) were women, and 62.3% (*n* = 81) had not seen anyone die, although 82.3% (*n* = 107) had suffered the loss of someone close and none had had any prior contact with the simulation classroom (Table 2).

As for the questionnaire for the evaluation of the EOL simulation project, the results showed that the content validity index (CVI) of the items calculated through the evaluation conducted by the six experts was 1, which indicates the maximum score, and that the items selected were considered essential by all the experts in the forms in which they were created. The internal consistency was α = 0.93. The questionnaire was completed by 130 students, and on the scale from 1 to 10, the mean score given to the project was 7.68 (SD = 1.10) (Table 3). For the different questions that comprised the questionnaire, the mean scores oscillated between 6.43 and 8.0. The highest rated activities were the discussion group and the simulation.

When comparing the mean scores of the two courses (2018–2019 vs. 2019–2020) (Table 3), significant differences were obtained in three items associated with the high-fidelity simulation. Accordingly, in the academic year 2019–2020, a significant increase was observed in the average score associated with the exploration of feelings and fears (t = −2.37, *p* = 0.019), the promotion of dialogue (t = −2.23, *p* = 0.028) and the acquisition of communication skills (t = −2.32, *p* = 0.022).

From the qualitative data, five themes emerged (Table A3): The dialogue about death; The professional management of the end of life; The communication with the patient; The management with the family; The teaching about the end of life.

### 3.1. The Dialogue about Death

The main concept of the reflection on death is to consider that death is a natural process, and to a lesser degree, to avoid turning it into a taboo subject.

The ideas expressed concerning the best way to die are focused on calmness, pain and company.

The greatest fear related with death was witnessing the pain, suffering and death of loved ones.

### 3.2. The Professional Management of the End of Life

The following ideas, which stand out for their frequency of coding, are related to professional practice and the EOL: Getting used to dealing with death; Learning to react to death; Fear of death harms professional practice.

### 3.3. Communication with the Patient

Another of the recurring themes was the valuing of the patient, and his or her sensitivity, as a fundamental component of communication. The issues of unawareness of the reactions of the patient, and patients who want to know the truth, stand out.

The second concept recorded was the lack of knowledge about the language of death, including the complexity of being prepared for informing about the EOL, which is the idea or reflection that was highlighted in the discourse of the participants.

### 3.4. Management with the Family

Despite the low frequency of coding, this was included in the results as it provided important topics, such as support for the deceased person or assessment of the patient’s family context along with the orientation during grieving.

### 3.5. Teaching about the End of Life

Opinions with respect to the usefulness of the training on EOL, the simulation and the dialogue, the novelty of the methodology employed, and the rest of the techniques utilized in the development of the teaching task, were also highlighted.

The highest scores were obtained for the simulation itself and the dialogue. Most highly rated was the value of experiencing the simulation, the opportunity to practice communication, the opportunity to practice patient assessment and the opportunity to learn how to show sensitivity. Realism was also highly rated.

The most notable issue arising from the simulation was related to the importance of participating in the simulation so that it truly has a learning effect on the participating students.

Other emerging subjects from the analysis were associated with participants’ concern about their classmates seeing the simulation, about the technique covering the entire teaching process or about the importance of having the support of a professor who “acts” as a family member during the course of the simulation.

With respect to the stories, the opinion of this resource, given by the participants, is that they favored the imagination, along with the idea that it is a learning task to express feelings.

As for the discussion groups, the most noticeable idea was the usefulness of the tool and the learning of other’s feelings, which was linked to two related ideas, including the importance of the respect of others and what the acquisition of different points of view had meant for the participants.

Another significant topic in the analysis was the positive character of the didactic approaches conducted with respect to the EOL. How to learn about what not to do, and more specifically, learning through mistakes, or avoiding making them, was another important observed subject. Likewise, but with less coding frequency, ideas related to the complementarity and the sequential nature of the activities, and the possibility of communicating and sharing information among students, were also stressed.

As for the suggestions for improvement proposed by the students, there seemed to be a general consensus that the project encourages collaborative work, and there was a suggestion of increasing the time dedicated to the simulation and broadening the methodology to other courses.

## 4. Discussion

The educational project we present is a dynamic and innovative initiative of EOL, in which the combination of different methodologies, such as the reading of children’s stories whose main theme is death, discussion groups and high-fidelity simulation, has made possible a sequential approach to the EOL for 130 students enrolled in their second year of the Nursing Degree at the University of Cantabria, who had not yet experienced clinical practice in real healthcare settings.

The project was well-evaluated by the students, especially the sessions dedicated to the dialogue and focused on the simulation. Furthermore, most of the students considered that it should continue to be taught in future academic years, and they even suggested increasing the number of simulation sessions.

The need for more training with relation to the final care of life has implications for the development of the curriculum in pre-graduate nursing programs, which have to provide the graduating nurses with the knowledge and skills needed to provide high quality care to the patients who are dying, as well as their families [40]. Nurses are the health professionals who are most closely in contact with individuals at the end of their lives, and they also play a vital role in providing care to their patients as well as their relatives. Despite contact with death and dying being common in a nurse’s work, many studies have shown that nursing students, as well as working nurses, experience a broad set of unpleasant feelings and emotions associated with death, such as fear, sadness, frustration and anxiety; they often do not feel sufficiently prepared to care for their patients and their families at the EOL, and often experience difficulties in coping with the death of their patients [3,4,5,41].

Teaching the nursing students to provide care at the EOL is important and challenging. Given that traditional in-person learning is being increasingly superseded by digital technologies, this provides an opportunity for the development of new forms of education concerning the EOL. Bayle et al. [42], in their study designed to examine the use of online technology for the improvement of the learning of nursing students about care at the EOL in the United Kingdom and the United States, recommended that teaching should be inclusive, focused and interesting, and should support individual collaborative and authentic learning. Bassah et al. [43], in a study that describes the experiences and perceptions of the nursing students in Cameroon concerning the strengths and weaknesses of a course on palliative care, concluded that the use of a variety of interactive educational strategies, including supervised clinical practice, is considered by the nursing students to be vital for improving learning in palliative care education.

Quality of care at the EOL requires effective communication skills [44,45]. However, the nursing students, in general, lack confidence, and have provided information regarding the limited opportunities for developing these skills. Simulation is a learning tool that allows one to develop, in a safe environment, such technical and non-technical skills as leadership, teamwork, task delegation and, of course, communication skills. Several studies have described the improvement of communication skills through simulation [46,47]. Simulation is an efficient medium for preparing the students to communicate with dying patients and their families, as the present study, as well as others, have shown [48].

End-of-life programs generally help students acquire communication skills, learn concepts, and improve the administration of this type of care. In addition, the students perceived the experience as an opportunity to learn more about oneself, gain trust and support critical thinking. Nonetheless, the evidence available in this field is limited, due to the small number of studies, plus the limited data reported. Thus, further studies on this subject are necessary.

### Limitations

This study has several strengths and limitations. This project allows a successive approach to the theme of the EOL, which, by combining different methodologies, is dynamic and well-received by students, especially the sessions dedicated to dialogue and simulation. Another strength is the combination of quantitative and qualitative data, allowing for the combination of both types of questioning techniques. There are also a number of limitations to be mentioned. These include the non-random selection of the sample and the participation of students from a single university and a single country, which is a source of bias that should be taken into consideration when interpreting the results and extrapolating them to other contexts. The use of indirect methods, such as questionnaires and scales, for evaluating the project can likewise be a source of bias, due to the subjectivity of this information. Additionally, the bias caused by social desirability should also be taken into account when interpreting the results. Lastly, the evaluation was conducted a month after the end of the sessions, and future research should consider repeating the measurements over a longer period of time in order to assess the preservation of the acquired communication skills in the medium- and long-term.

## 5. Conclusions

High-fidelity simulation allows the development of communicative skills related to EOL care in novice nursing students. The increased time spent on high-fidelity simulation has significantly favored the exploration of feelings and fears about death, has encouraged dialogue about the EOL, and has led to the acquisition of communication skills instrumental to facing death and dying.

## Figures and Tables

**Table 1:** Sessions of the innovation project (number, duration and tasks) with an indication of the main actor (teacher/student).

SES *n*	Duration	Objective	Task	
			Teacher	Student
1	30′	To get 2nd grade nursing students involved in the project	To present the project and request participation	To read the information sheet and sign, if desired, the informed consent
2	60′	To review theoretical concepts related to the end of life	To guide and supervise the performance of tasks	To watch videos and participate in individual and group activities
3	120′	To facilitate reflection on and approach to the issue of the end of life	To organize and guide the activities of approach and reflection	To select and read one story, and discuss the readings
4	60′	To encourage dialogue on the end of life	To moderate and lead the dialogue	To participate in a discussion group
5	120′–360′ *	To practice behaviors in simulated end-of-life situations	To guide and act in the simulation sessions and to collect information that allows a later evaluation. To lead the debriefing to highlight the importance of communication, emotional skills and active listening	To prepare and participate in high-fidelity simulation scenarios. To practice communication and emotional skills when facing death and dying in a safe environment
6	60′	To assess the project	To analyze the students’ behavioral and communication skills, as well as the information provided about the project	To complete the evaluation questionnaire

* In the academic year 2018–2019 there were 2 sessions of high-fidelity simulation (120 min), and in 2019–2020 this was extended to 6 sessions (360 min). SES: Session.

**Table 2:** Closed-ended questions.

Questions	Global (*n* = 130)				2018–2019 (*n* = 70)				2019–2020 (*n* = 60)				*p* ^a^
	Yes	%	No	%	Yes	%	No	%	Yes	%	No	%	
Have you seen someone die?	49	37.7	81	62.3	27	38.6	43	61.4	22	36.7	38	63.3	0.823
Have you suffered the death of someone close?	107	82.3	23	17.7	64	91.4	6	8.6	43	71.7	17	28.3	0.003
Do you fear facing death during your clinical practices?	78	60.0	52	40.0	38	54.3	32	45.7	40	66.7	20	33.3	0.151
Would you like to work in palliative care?	48	36.9	82	63.1	29	41.4	41	58.6	19	31.7	41	68.3	0.250
Do you think Nursing students should receive training related to death?	130	100			70	100			60	100			

^a^ Chi squared.

**Table 3:** Evaluation of the project.

Questions	Global (*n* = 130)		2018–2019 (*n* = 70)		2019–2020 (*n* = 60)		*p* *
	Mean	SD	Mean	SD	Mean	SD	
The tales							
They increased your sensibility about the subject of end of life.	6.7	1.8	6.8	1.8	6.7	1.8	0.695
They made you reflect about end of life.	7.0	1.8	7.1	1.6	6.9	1.9	0.717
They favored the exploration of your feelings and fears about end of life.	6.5	1.8	6.4	1.9	6.6	1.7	0.497
They fomented your dialogue about the end of life.	6.5	1.8	6.6	1.8	6.5	1.9	0.666
They favored the acquisition or strengthening of your communication skills to cope with the end of life.	6.4	1.8	6.4	1.8	6.5	1.8	0.724
They increased your sensibility about the subject of end of life.	7.4	1.7	7.6	1.6	7.2	1.8	0.336
They made you reflect about end of life.	7.7	1.5	7.7	1.4	7.6	1.6	0.698
They favored the exploration of your feelings and fears about end of life.	7.5	1.6	7.6	1.4	7.4	1.7	0.577
They fomented your dialogue about the end of life.	7.5	1.7	7.5	1.8	7.4	1.6	0.860
They favored the acquisition or strengthening of your communication skills to cope with the end of life.	7.2	1.6	7.3	1.6	7.1	1.6	0.569
They increased your sensibility about the subject of end of life.	7.4	1.5	7.2	1.5	7.6	1.5	0.141
They made you reflect about end of life.	7.5	1.5	7.3	1.5	7.8	1.5	0.118
They favored the exploration of your feelings and fears about end of life.	7.4	1.6	7.0	1.6	7.7	1.6	0.019
They fomented your dialogue about the end of life.	7.3	1.7	7.0	1.8	7.7	1.6	0.028
They favored the acquisition or strengthening of your communication skills to cope with the end of life.	7.7	1.5	7.4	1.3	8.0	1.7	0.022
Global							
From 1 to 10, what score would you grant the project?	7.7	1.1	7.7	1.1	7.7	1.1	0.892

SD: standard deviation; * t Student test.

## Appendix A

**Table A1:** List of selected Tales. Published in Spanish, in paper format, 1984–2011.

Title in Spanish (Title in English)	Author	Year of First Edition	Publishing House
Abuela de arriba, abuela de abajo (Nana Upstairs and Nana Downstairs)	Tomie de Paola	1994	SM
Abuelo, ¿dónde estás? (Grandfather, where are you?)	Elisa Mantoni	2006	Everet
¡Buenas noches abuelo! (Good-night gradpa!) *	Roser Bausà and Carme Peris	2004	Lóguez
¿Cómo es posible??! La historia de Elvis (How is it possible??! The story of Elvis)	Peter Schössow	2005	Lóguez
Cuando estoy triste (Getting out of stress mess!)	Michaelene Mundy and RW Alley	1998	San Pablo
¿Dónde está el abuelo? (Where’s Grandpa)	Mar Cortina and Amparo Peguero	2001	Tandem
¿Dónde está güelita Queta? (Where is grandma Queta?) *	Nahir Gutiérrez and Alex Omist	2011	Destino
El abuelo de Tom ha muerto (Tom’s grandfather has died)	Marie Aline Bawin and Colette Helling	2000	Combel
El ángel del abuelo (Granpa’s angel)	Jutta Bauer	2001	Lóguez
El árbol de los recuerdo (The tree of memories) *	Britta Teckentrup	2013	Nubeocho
El cuento de Thumpy (Thumpy’s Story)	Nancy C. Dodge and Kevin Veara	1984	Share Pregnancy & Infant Loss Support
El jardín de mi abuelo (My grandfather’s garden) *	Mª Ángels Gil and Mabel Piérola	2007	Bellaterra
El libro triste (Michael Rosen’s Sad Book)	Michael Rosen and Quentin Blake	2004	Serres
El mejor truco del abuelo (Gran-gran’s best trick)	L. Dwight Holden and Michael Chesworth	1989	Magination Press
El niño de las estrellas (Kid of the stars)	Patrik Somers and Katrien van der Grient	2000	ING edicions
El pato y la muerte (Duck, Death and the Tulip)	Wolf Erlbruch	2007	Barbara Fiore Editora
Julia tiene una estrella (Julia has a star)	Eduard José and Valentí Gubianas	2006	La Galera
La caricia de la mariposa (The caress of the butterfly) *	Christian Voltz	2008	Kalandraka
¡Mamá! (Mom!) *	Iñaki Zubeldia	2006	edebé
Más allá del gran río (Beyond the great river)	Armin Beuscher and Cornelia Haas	2004	Juventud
Mi abuelo era un cerezo (My Grandfather was a Cherry Tree)	Angela Nanetti	2001	SM
Mi abuelo es una estrella (My grandpa is a star) *	Sacha Azcona	2016	Bruño
Mi amigo el Sauce (Gentle Willow)	Joyce C. Mills and Cary Pillo	2008	Sana colita de rana
Nana vieja (Old Pig)	Margaret Wild and Ron Brooks	1995	Ekaré
No es fácil pequeña ardilla (It’s not easy little squirrel)	Elisa Ramón and Rosa Osuna	2003	Kalandraka
Osito y su abuelo (Little Bear’s Grandad)	Nigel Gray and Vanessa Cabban	1999	Timun Mas
Para siempre (Always and Forever)	Alan Durant and Debi Gliori	2004	Timun Mas
¿Qué viene después del mil? (What comes after a thousand?) *	Anette Bley	2009	Takatuka
Te echo de menos (I miss you)	Paul Verrept	1998	Juventud
Yo las quería (I wanted them) *	María Martínez and Carme Solé	1984	Destino

* At the time of this study the tale was not available in English; therefore, the title is a translation by the authors of the study.

**Table A2:** Focus group guide.

Do you think it is useful to include this type of teaching about the end of life in the teaching of the degree? Why?
In relation to the training you have received, we are interested about how the different activities you have conducted have had an influence, or how you evaluate them.
In relation to what you have commented until now, do you think the training you have received related to the education about the end of life with the methodology employed, will help you in the future as professionals differently, as compared to traditional methodologies (lecture and written exam…)? Why?
Do you think this training with the methodologies employed should still be taught to students enrolled in the second year of the Nursing degree? Why?
Would you suggest some aspects of improvement for the teaching of future courses? What would you suggest and why?

**Table A3:** Themes and verbatim extracts.

Themes	Verbatim Extract
Dialogue about death	“It is natural life process, and one has to think of it that way”
	“With a clear conscious, surrounded by loved ones and without pain”
	“Without pain, and with people you want by your side”
	“That it is a painful process or longer than necessary”
	“That the people I love the most die”
	“Watch people close to me suffer, not knowing how to deal with the lack of that person”
The professional management of the end of life	“Because I see it as something natural, as something that no one can escape and something that will happen to all of us”
	“I think that although at first it will be difficult, we should be conscious that it is something that will occur and that we should learn to cope with it the best way possible”
	“Because I do not like to see people suffer, nor patients or family members”
	“Because I don’t know if I could deal with it, and I think it is very hard work for which one has to be psychologically prepared”
	“To empathize a lot with a patient and that later will affect my personal life”
Communication with the patient	“Because I would like to accompany the patients until their death, it is not easy to face it alone”
	“To help as much as possible in the last moments”
	“That I will not know how to cope with the situation. For example, that I cry and not know what to say in that moment”
	“No one is ready to say this: you are going to die”
	“You are not going to know what to say, no matter how professional you are or how many practicals you have done”
Management with the family	“ It has helped me a lot to know that we really have to do when we have to face a situation in which we have a patient or family members who are going to face death”
Teaching about the end of life	”To know how to deal with it”
	“Because it is the day-to-day in our profession, we are always going to be in direct contact with the people, disease and death, and it is necessary to know how to approach it”
Other emerging themes: Simulation, clinic and dialogue	“The high-fidelity simulation, because it is the activity that closely resembles the future clinical practice. It offers the possibility to reflect and discover coping strategies when attending the patient and the family members”
	“The dialogue in a group, as one loses some of the fear when talking about death and one can see how the others cope with this process”
	“The simulation because it is more realistic”
	“The simulation, because you really find yourself facing a situation of death”
	“I value the dialogue the most, as you know the perspectives of the others and it makes you reflect more about the subject”
	“That the simulation session last for a greater number of sessions so that more people could do it”
	“Increase the simulations”
